# Thalamus exhibits less sensory variability quenching than cortex

**DOI:** 10.1038/s41598-019-43934-9

**Published:** 2019-05-20

**Authors:** E. Poland, T. H. Donner, K. -M. Müller, D. A. Leopold, M. Wilke

**Affiliations:** 1 Department of Cognitive Neurology, UMG, University Medicine Goettingen, Robert-Koch-Str. 40, Goettingen, 37075 Germany; 2 University Medical Center Hamburg-Eppendorf, UKE, Department of Neurophysiology and Pathophysiology, Building N43, Martinistr. 52, 20246 Hamburg, Germany; 3 Consumer Behavior, HFU Business School, Jakob-Kienzle-Str. 17, 78054 Villingen-Schwenningen, Germany; 4 Section on Cognitive Neurophysiology and Imaging, Laboratory of Neuropsychology, National Institute of Mental Health, National Institutes of Health, Building 49, Room B2J-45, MSC-4400 49 Convent Dr., Bethesda, MD 20892 USA; 5 DFG Center for Nanoscale Microscopy & Molecular Physiology of the Brain (CNMPB), Robert-Koch-Str. 40, Göttingen, 37075 Germany; 6 German Primate Center, DPZ, Leibniz Institute for Primate Research, Kellnerweg 4, Goettingen, 37077 Germany

**Keywords:** Thalamus, Computational neuroscience

## Abstract

Spiking activity exhibits a large degree of variability across identical trials, which has been shown to be significantly reduced by stimulus onset in a wide range of cortical areas. Whether similar dynamics apply to the thalamus and in particular to the pulvinar is largely unknown. Here, we examined electrophysiological recordings from two adult rhesus macaques performing a perceptual task and comparatively investigated trial-to-trial variability in higher-order thalamus (ventral and dorsal pulvinar), the lateral geniculate nucleus (LGN) and visual cortex (area V4) prior to and following the presentation of a visual stimulus. We found spiking variability during stable fixation prior to stimulus onset to be considerably lower in both pulvinar and the LGN as compared to area V4. In contrast to the prominent variability reduction in V4 upon stimulus onset, variability in the thalamic nuclei was largely unaffected by visual stimulation. There was a small but significant variability decrease in the dorsal pulvinar, but not in the ventral portion of the pulvinar, which is closely connected to visual cortices and would thus have been expected to reflect cortical response properties. This dissociation did not stem from differences in response strength or mean firing rates and indicates fundamental differences in variability quenching between thalamus and cortex.

## Introduction

Cortical activity is characterized by a large degree of variability^1–3^ that poses challenges for relating changes in neural activity to stimulus conditions and behavioral states^4^. At the same time, neural variability itself is increasingly used to infer neurocomputational principles and to assess neural integrity in patient populations^5,6^.

On the level of neuronal spiking, variability across trials, which is typically measured as the mean-corrected firing rate variance (Fano factor), is thought to arise in large part from widespread fluctuations in cortical excitability^7–10^. It has been well-established that the onset of a stimulus results in a reduction of trial-to-trial variability, often referred to as variability quenching, that is not a trivial by-product of changing firing rates and thought to constitute a common property of a wide range of cortical areas^1^. Variability quenching occurs even among neurons that do not exhibit firing rate changes upon stimulus onset, suggesting that the variability decline does not depend on the response properties of individual neurons^1,10^. Since extensive variability in neural responses limits the reliability with which information can be encoded, its stimulus-driven reduction can be considered to improve sensory processing^11,12^.

Whether equivalent stimulus-induced decreases of spiking variability exist in the thalamus is still largely unclear. Two major thalamic nuclei are closely related to visual cortex: the lateral geniculate nucleus (LGN) and the pulvinar^13^. The LGN is considered a ‘first-order’ nucleus as it receives driving input from the retina and projects to layer 4 of cortical area V1^14^. The pulvinar is considered a ‘higher-order’ nucleus which receives major driving input from layer 5 of cortex and participates in cortico-thalamo-cortical pathways^15,16^.

While the retinotopically organized ventral pulvinar portion receives input from the superior colliculus and primarily exchanges connections with striate and extrastriate visual cortices, its non-retinotopic dorsal portion primarily interconnects with ‘associative’ cortices such as superior temporal, posterior parietal, and prefrontal cortices^13,17–19^. Pulvinar neurons in both portions are visually responsive and often modulated by eye movements and internal variables such as visual attention and perception^20–24^. Both pulvinar portions exchange connections with mid-level visual area V4^25^, and sensory processing in the pulvinar is conceived as a reflection of its cortical inputs as V4 and pulvinar response properties are similar^26^. Interactions between the pulvinar and V4 have been shown to be necessary for visual and attentional processing^23,26^.

While cortical variability has been extensively studied, few studies have investigated this dimension in the thalamus. Previous studies mainly focused on comparing response variability between visual cortex and first-order sensory thalamus and consistently found LGN responses to be less variable than cortical responses^8,10,27,28^. Regarding the pulvinar the evidence is less clear: In one study response variability in the pulvinar has been found to be lower than in visual cortices^27^ while another study in anesthetized ferrets reported higher trial-to-trial variability for the lateral-posterior pulvinar complex during visual stimulation compared to V1^29^. This discrepancy between the two studies could be attributed to differences in cortical state as higher variability may occur in anesthetized animals when large amplitude, low frequency fluctuations become more prevalent^10^, but warrants further investigation. Moreover, a systematic investigation of the presence of a quenching effect in the thalamus, and in particular in higher-order thalamic nuclei that exchange recurrent connections with the cerebral cortex such as the pulvinar, is still lacking.

The aim of the present study was to examine whether trial-to-trial variability dynamics in the thalamus are fundamentally similar to those in cortex and show an equivalent reduction upon visual stimulation. To investigate this question we compared spiking variability from neurons in dorsal and ventral pulvinar, LGN and area V4 recorded simultaneously in two macaque monkeys performing the same perceptual task. We found LGN and pulvinar activity to be considerably less variable than V4 activity even prior to stimulus onset, while visual stimulation did not reduce thalamic variability to a similar extent as in cortex.

## Results

### Spiking variability following visual stimulation

**Figure 1:**
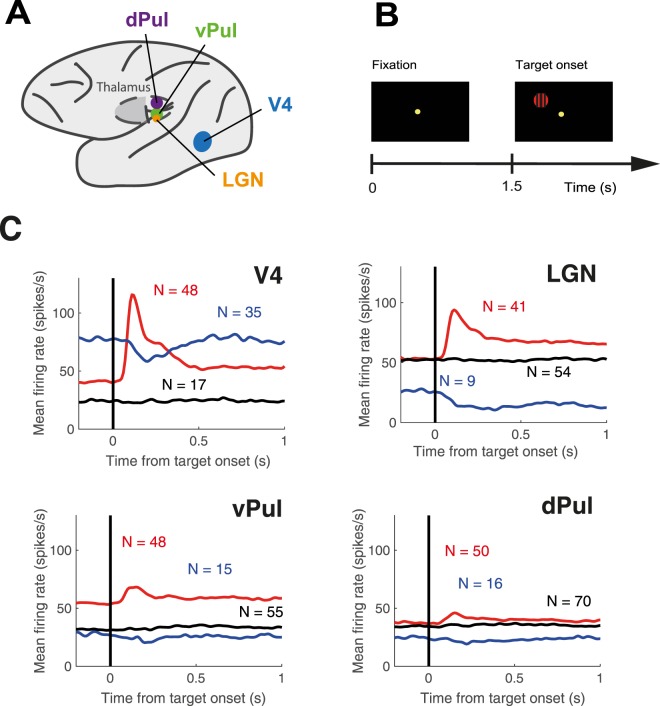
Recording sites and stimuli. (**A**) Schematic of the recording sites in monkeys E and B: and V4 (blue), ventral pulvinar (vPul, green), dorsal pulvinar (dPul, purple), lateral geniculate nucleus (LGN, orange). MRI-based reconstructions of the thalamic recording sites can be found in^24^. (**B**) Visual stimuli were presented through a mirror stereoscope. Each trial began with acquisition of the central fixation spot. 1.5 s later the target stimulus (red disk or grating) was shown. Target stimuli were shown to the left eye. Monkeys were required to pull the lever upon target presentation and to continue holding it as long as the target was visible. Trial intervals of interest were the pre-stimulus fixation period 1000–500 ms prior to target onset and visual stimulation 300 ms pre −300 ms post target stimulus. **(C)** Mean firing rates of sites that showed significant rate increases (red), significant rate decreases (blue) or no significant responses to the onset of the target stimulus (black) for each brain region.

To assess whether the well-established cortical decrease of trial-to-trial variability upon stimulus onset applies to the thalamus, we first examined the effect of visual stimulation on neural trial-to-trial variability of single- and multi-unit activity recorded in area V4 (81 MUA, 19 SUA), the ventral (108 MUA, 12 SUA) and dorsal pulvinar (102 MUA, 34 SUA) and the LGN (89 MUA, 16 SUA) of two rhesus macaques performing a detection task (Fig. 1A). While the main focus of the current study was trial-to-trial variability in the pulvinar nuclei, we have included the LGN data as a reference point based on previous literature^8,10,27,28^. The data of the two monkeys were very similar (see Supplementary Information Fig. S1 for a separate analysis) and thus pooled. The presentation of the target stimulus (Fig. 1B) elicited significant responses in 83 out of 100 sites in area V4, 63 out of 118 sites in the ventral pulvinar, 66 out of 136 sites in the dorsal pulvinar and 50 out of 105 sites in the LGN (Wilcoxon rank-sum test, p < 0.05, summary Fig. 1C).

**Figure 2:**
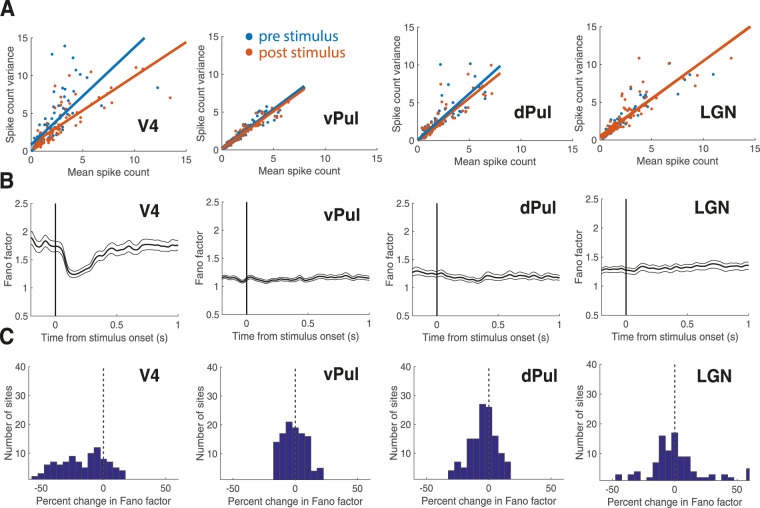
Trial-to-trial variability decrease following visual stimulation. **(A)** Mean spike count and variance of spike counts during the pre-stimulus window 300 ms before target onset (blue) and the post- stimulus window 300 ms after target onset (orange) for individual sites regardless of responsiveness (V4 N = 100, vPul N = 118, dPul N = 136; LGN N = 105). Lines represent the least squares fit. **(B)** Average of raw Fano factors aligned to the time of target stimulus onset (0 s) for complete populations (V4 N = 100, vPul N = 118, dPul N = 136, LGN N = 105) calculated with a 50 ms sliding window. Error bars indicate +/− 1 SEM. **(C)** Population histograms (V4 N = 100, vPul N = 118, dPul N = 136; LGN N = 105) of percent change in Fano factor from the pre to post stimulus window.

Figure 2A shows the raw mean spike count and spike count variance across trials 300 ms prior to (blue) and 300 ms post target onset (orange) for all sites independent of responsiveness to the visual target. As the Fano factor denotes the variance to mean ratio of spike counts, the slopes of the least squares lines in Fig. 2A represent trial-to-trial variability within the examined population before (blue) and after stimulus presentation (orange). The estimates based on the slopes of the least-squares lines showed a substantial variability decrease with stimulus onset in area V4, whereas pre and post stimulus slopes in the thalamic pulvinar and LGN sites were similar.

The average time course of trial-to-trial variability around the onset of the target stimulus is shown in Fig. 2B. As expected from the literature^1,30,31^, mean trial-to-trial variability in area V4 decreased substantially following onset of the target stimulus. In contrast to cortical activity, trial-to-trial variability in the thalamic nuclei was largely unaffected by the visual stimulus presentation. Comparing the average Fano factors of the 300 ms pre and 300 ms post target onset intervals in each brain region with Wilcoxon signed-rank tests (Table 1), we observed the expected significant decrease in Fano factor following stimulus onset in area V4 (mean change −16%, p = 6.91e-11). Spiking variability was unaffected in the ventral pulvinar (mean change 0%, p = 0.29) and LGN (mean change 2%, p = 0.55) and only marginally, albeit statistically significantly reduced in the dorsal pulvinar (mean change −4%, p = 1.72e-05).

**Table 1:** Summary of mean Fano factors +/− standard deviation 300 ms pre and 300 ms post onset of the target stimulus. N denotes the number of recording sites.

N	V4	vPul	dPul	LGN
	100	118	136	105
mean Fano factor pre stimulus +/− SD	1.80 +/− 0.98	1.13 +/− 0.25	1.26 +/− 0.59	1.28 +/− 0.45
mean Fano factor post stimulus +/− SD	1.41 +/− 0.57	1.12 +/− 0.25	1.19 +/− 0.51	1.29 +/− 0.51

Figure 2C shows the distribution of percent change in Fano factor from pre to post stimulus intervals for all recorded sites. While the V4 population shows a clear leftward shift indicating decreased variability following target onset in the majority of individual sites, this shift appears absent in ventral pulvinar and LGN populations and is far less obvious in the dorsal pulvinar. Accordingly, the percent change in Fano factor was significantly greater in area V4 than in ventral and dorsal pulvinar and the LGN (Two-sample t-tests, V4-vPul p = 1.25e-12, V4-dPul p = 2.80e-08, V4-LGN p = 7.14e-08). Response variability, that is variability during the considered post-stimulus window (Table 1), was significantly lower in both pulvinar sub-nuclei than in area V4. Similarly, LGN responses were also less variable than those of V4 but not significantly so (Wilcoxon ranked-sum tests, V4-vPul: p = 9.36e-05, V4-dPul: p = 4.11e-05, V4-LGN p = 0.09). A separate examination of SUA and MUA yielded very similar results as we observed a significant quenching effect in both V4 SUA and MUA that was also significantly larger than variability changes in the thalamic regions. The variability decrease in the dorsal pulvinar was only significant in the multi-unit data, which may however be due to the low sample size for single-units (for details see Supplementary Information Fig. S2).

Since the Fano factor measures the degree of trial-to-trial variability within single neurons or multi-units, it does not by itself provide information as to whether the variability is independent or shared across many neurons. We thus additionally sought to examine correlated variability within and between area V4 and the pulvinar regions by calculating the spike count (noise) correlations in simultaneously recorded unit pairs. Calculations of spike count correlations based on multi-unit data can lead to a consistent overestimation of the correlation strength^32^, which does however not affect the relative comparison of correlations before and after stimulus onset. Within area V4 shared variability also substantially decreased with the onset of the target stimulus (Supplementary Information Fig. S3). We observed no significant change of correlated variability with stimulus onset in the pulvinar, nor between pulvinar and V4.

### Relationship to stimulus-induced changes in firing rate

**Figure 3:**
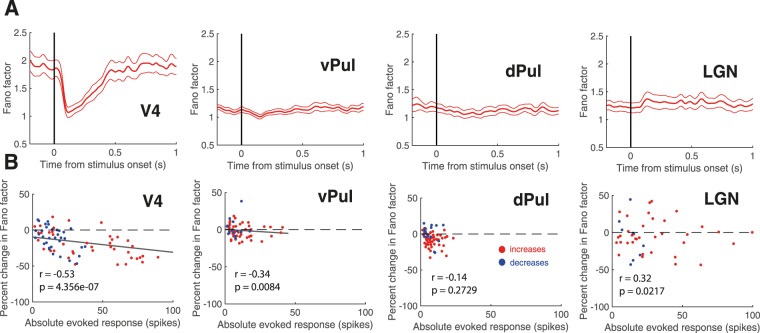
Dependence on the visually evoked response strength. (**A**) Fano factor around the time of target onset for the subsets of visually responsive sites that responded with significant rate increases (V4 N = 48, vPul N = 48, dPul N = 50, LGN N = 41). Error bars indicate +/− 1 SEM. **(B)** Percent change in Fano factor from the 300 ms pre to 300 ms post stimulus interval as a function of the evoked response strength (absolute spike difference) for sites with significant rate increases (red) and decreases (blue).

Given this prominent difference among cortical and thalamic populations with respect to the stimulus-induced variability decline, we next asked whether changes in firing rate might impact variability in cortical and thalamic neurons differently. We examined whether the observed quenching differences between V4 and the thalamic sites could be attributed to differences in the strength of the visually evoked responses. As the pulvinar populations in our data set had a larger percentage of non-responsive sites and responses were generally less pronounced than in area V4 (Fig. 1C), we wondered whether the minimal stabilization effects in the thalamic regions were due to differences in response properties of the examined samples. We thus examined the time courses of trial-to-trial variability around the time of target stimulus onset for the subset of sites which showed positive significant visually evoked responses (V4 N = 48, vPul N = 48, dPul N = 50, LGN N = 41, Fig. 3A) and found them to be very similar to the behaviour of the complete population described above. The percent change distribution from pre to post stimulus interval was significantly different from zero in area V4 and the dorsal pulvinar, but not in the ventral pulvinar portion or in the LGN (One-sample t-tests, V4 p = 2.57e-11, vPul p = 0.02, dPul p = 3.05e-06, LGN p = 0.32). Figure 3B shows the percent change in Fano factor as a function of the absolute evoked response strength for individual visually responsive sites. In V4, we found a strong negative correlation between the decrease in Fano factor and the strength of the evoked response (Pearson’s correlation coefficient after outlier correction r = −0.53, p = 4.36e-07), suggesting that the variability reduction may have been at least in part due to the rate change itself. This negative correlation was present in sites that responded with rate increases (N = 48, r = −0.53, p = 9.45e-05, red) as well as in sites that responded with a rate decrease (N = 35, r = −0.54, p = 0.10e-2, blue). In the ventral pulvinar we observed a significant negative correlation between the variability decline and the visually evoked response strength (r = −0.34, p = 0.84e-2), while there was no significant correlation after Bonferroni correction in the dorsal pulvinar (r = −0.14, p = 0.27) or in the LGN (r = 0.32, p = 0.02).

**Figure 4:**
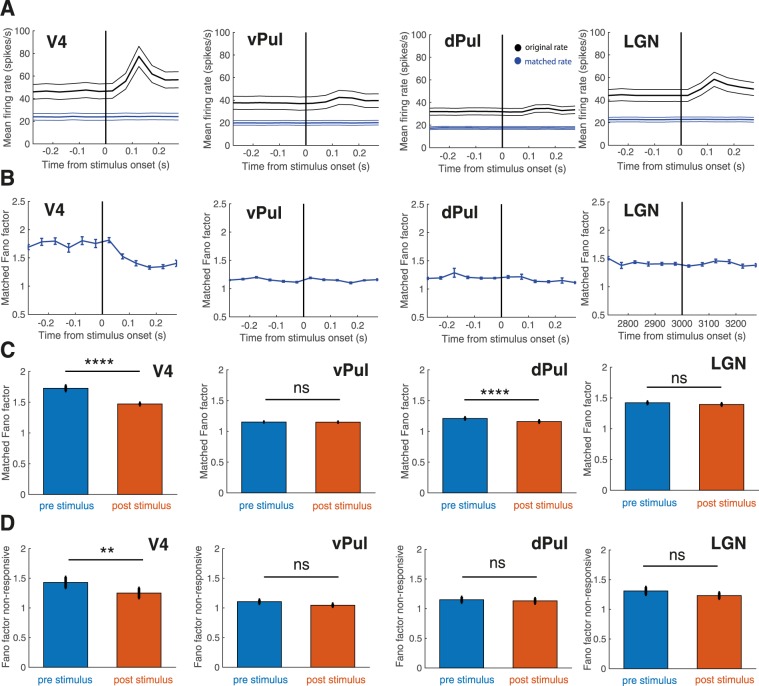
Controls for changes in firing rate. (**A**) Raw (black, V4 N = 100, vPul N = 118, dPul N = 136, LGN N = 105) and distribution-matched mean firing rates (blue, V4 N = 43, vPul N = 60, dPul N = 79, LGN N = 60) around the time of stimulus onset. Error bars show +/− 1 SEM. **(B)** Distribution-matched Fano factor computed from the data points representing the matched rate (V4 N = 43, vPul N = 60, dPul N = 79, LGN N = 60). Error bars indicate +/− 1 SEM. **(C)** Mean distribution-matched Fano factor for the 300 ms pre (blue) and 300 ms post stimulus interval (orange). Error bars indicate +/− 1 SEM. Wilcoxon signed-rank tests, Bonferroni corrected, ****p < 0.0001. **(D)** Fano factor calculated based on non-responsive sites (Wilcoxon ranked-sum tests, p > 0.05; V4 N = 47, vPul N = 79, dPul N = 95, LGN N = 79) for 300 ms pre (blue) and 300 ms post RDM stimulus (orange). Error bars indicate +/− 1 SEM. Wilcoxon signed-rank tests, Bonferroni corrected, **p < 0.01.

Due to the dependence on the visually evoked response we sought to exclude that the observed variability decline was primarily caused by rising and falling rates in response to the target stimulus by matching firing rate distributions over the 300 ms to 300 ms post stimulus period equivalent to the approach developed in a previous study^1^.The original mean firing rate (black) as well as the matched firing rate (blue) around the time of stimulus onset are shown in Fig. 4A. The firing rates obtained through the matching procedure were comparable between regions (24 spikes/s in V4, 20 spikes/s in the ventral pulvinar, 19 spikes/s in the dorsal pulvinar and 23 spikes/s in the LGN). We then performed the equivalent statistical analysis on the pre and post target intervals using the distribution-matched values (Fig. 4B,C). Despite mean firing rates being kept constant over time, the stimulus-evoked variability decline remained significant in V4 and in the dorsal pulvinar, while again not being significant in the ventral pulvinar or in the LGN (Wilcoxon signed-rank test, V4: N = 43, p = 3.06e-07, vPul: N = 60, p = 0.55, dPul: N = 79, p = 5.33e-07, LGN: N = 60, p = 0.08), thus confirming the results obtained in the analysis of the full data.

While the Fano factor can be reliably computed based on multi-unit recordings due to the fact that the sum of independent Poisson-distributed values is itself Poisson-distributed, the success of the mean-matching procedure may not be guranteed^1^. As Churchland *et al*. have previously shown^1^, variability decreases with stimulus onset can also be observed in neurons that do not themselves respond to the stimulus. This allowed us to investigate the quenching effect under non-responsive conditions when the firing rate of the examined sites changes little. The target stimulus elicited significant responses in most V4 sites (Fig. 1C). We thus examined presentations of a large full-field random dot motion (RDM) pattern, which did not evoke significant visual responses in a large portion of sites (Wilcoxon rank-sum test, p > 0.05). Consistent with previous findings^1^ we observed a significant decrease in trial-to-trial variability following the onset of the motion stimulus in area V4 even when the firing rate did not change (Wilcoxon signed-rank test, N = 47, p = 6.53e-3, Fig. 4D). While variability in non-responsive sites decreased slightly throughout all regions, this effect was insignificant in non-responsive ventral pulvinar, dorsal pulvinar or LGN sites (Wilcoxon signed-rank tests, vPul: N = 79, p = 0.08; dPul: N = 95, p = 0.48, LGN: N = 79, p = 0.14), further confirming that the differences between thalamic and cortical regions in quenching behavior did not stem from differences in responsiveness. In addition to changes in firing rate, small eye movements may in principle impact quenching. We thus performed control analyses to account for this possibility and could confirm that the observed differences between regions did not stem from changes in microsaccade rate (Supplementary Information Fig. S4).

### Variability changes in the dorsal pulvinar

We were surprised to find a small but significant variability decrease following stimulus onset in the dorsal pulvinar but not in the ventral pulvinar portion, despite the latter being closely interconnected with the visual system and likely receiving inputs from area V4^18,33^. Since monkeys were required to pull the lever in response to the onset of the target stimulus and variability changes have been related to motor preparation^34^, we wondered whether the lever action itself might have contributed to the reduction of the Fano factor in the dorsal pulvinar, where neuronal firing is also related to hand movements^35^. We thus undertook a closer inspection of the RDM stimulus, which was not followed by a lever response. This had the additional benefit of allowing us to determine whether the different quenching behaviour in cortex and thalamic regions was reproducible with a different stimulus type.

**Figure 5:**
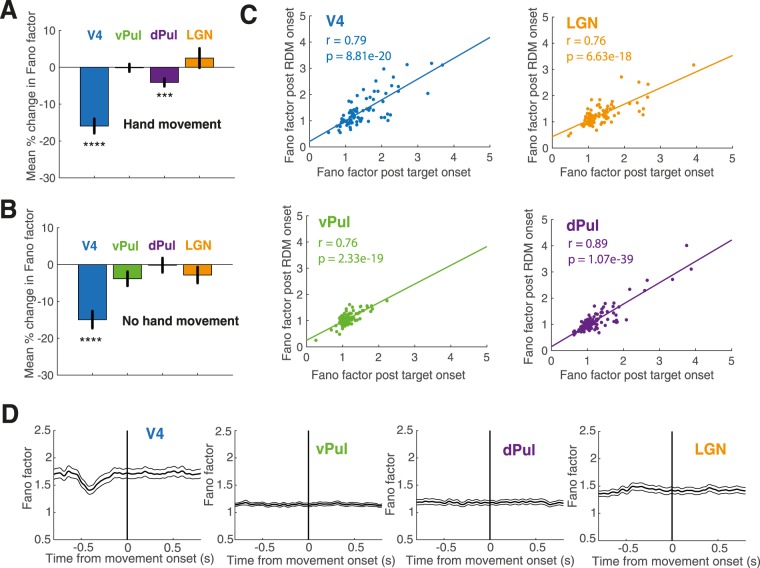
Quenching effect in the dorsal pulvinar and relation to hand movement. (**A**) Mean percent change in Fano factor from 300 ms pre to 300 ms post target stimulus intervals in area V4, the ventral and dorsal pulvinar and the LGN (V4 N = 100, vPul N = 118, dPul N = 136; LGN N = 105). Error bars indicate +/− 1 SEM. One-sample t-tests, ****p < 0.0001, ***p < 0.001. Upon target presentation, the animal was instructed to pull a lever. (**B**) Mean percent change in Fano factor from 300 ms pre to 300 ms post RDM stimulus intervals in area V4, the ventral and dorsal pulvinar and the LGN. Error bars indicate +/− 1 SEM. One-sample t-tests, ****p < 0.0001. The RDM stimulus was not followed by a hand movement. **(C)** Fano factors for the 300 ms post stimulus interval for the target and the RDM stimulus. Pearson’s correlation coefficients. **(D)** Fano factor time courses of data triggered to the time of the lever pull following the presentation of the target stimulus.

As for the target stimulus, we found a substantial decrease in Fano factor following the onset of the RDM stimulus in V4, whereas stimulus-induced changes in trial-to-trial variability in the pulvinar and LGN were again of smaller magnitude. Figure 5A,B summarizes the findings for the two stimuli. In contrast to the target stimulus onset with associated lever response (Fig. 5A), the full-field motion stimulus did however not elicit a significant variability decrease in the dorsal pulvinar portion (Fig. 5B), while area V4 continued to exhibit a significant stimulus-induced variability decline (One-sample t-tests, V4 p = 3.30e-09; vPul p = 0.05; dPul p = 0.93; LGN: p = 0.19). We compared Fano factor values of the 300 ms post stimulus interval for the target and the RDM stimulus within the common subset of recording sites and found response variability to be highly correlated between stimulus types in all examined regions (Pearson’s correlation coefficients, Fig. 5C).

We thus wondered whether the significant variability decrease in the dorsal pulvinar we previously observed in the presence of a lever response was due to the hand movement and examined trial-to-trial variability triggered to the time the animals pulled the lever. 32 out of 136 dorsal pulvinar sites showed significant motor responses (Wilcoxon rank-sum test, p < 0.05). There was no significant difference between Fano factors 300 ms prior and 300 ms following the lever response in the dorsal pulvinar (Wilcoxon signed-rank test, N = 136, p = 0.14) and Fano factor time courses triggered to the lever action were flat (Fig. 5D), suggesting that the small but significant variability decline following the target stimulus was not due to the movement itself, but may nonetheless have been related to motor preparation as it was no longer present in the absence of a subsequent motor response.

### Spiking variability prior to stimulus onset

In examining the effect of visual stimulation on trial-to-trial variability, we noted that neural activity was already considerably less variable in the pulvinar and the LGN than in area V4 prior to stimulus onset (Fig. 2B). This led us to wonder whether the absence of quenching in the pulvinar and LGN compared to cortex was related to thalamic spiking variability already being relatively low when sites were not directly driven by the onset of a stimulus. We thus examined a 500 ms period during stable fixation that began 500 ms after the acquisition of the fixation spot and ended 500 ms before the onset of the target stimulus (see Fig. 1B) in order to determine whether the degree of baseline variability was similar among the different regions.

**Figure 6:**
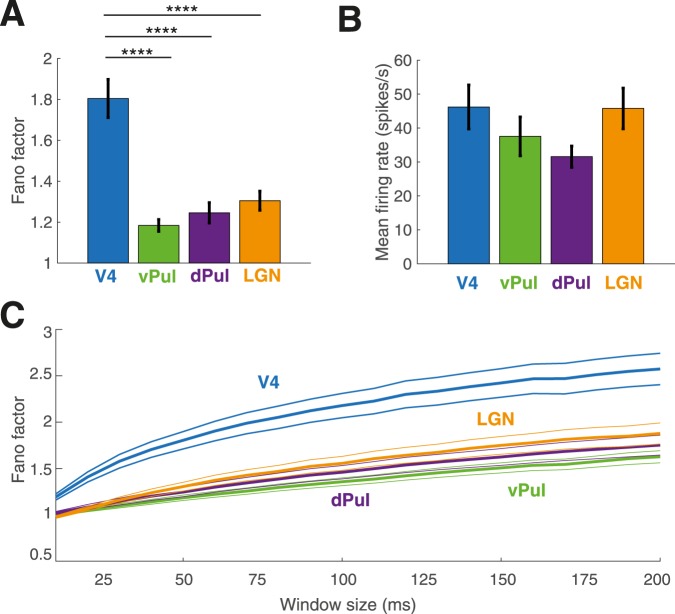
Trial-to-trial variability differences prior to stimulus onset. (**A**) Mean Fano factor calculated using non-overlapping 50 ms windows during the time interval of interest per region (V4 N = 100, vPul N = 118, dPul N = 136, LGN N = 105). Error bars indicate +/− 1 SEM. Wilcoxon signed-rank tests, Bonferroni corrected, ****p < 0.0001. (**B**) Mean firing rates during the ongoing activity interval as a function of brain region (V4 N = 100, vPul N = 118, dPul N = 136, LGN N = 105). **(C)** Fano factor as a function of counting window size in all brain regions (V4 N = 100, vPul N = 118, dPul N = 136, LGN N = 105). Error bars indicate +/− 1 SEM.

In the absence of changes in visual input, spiking activity was considerably more variable in area V4 than in the thalamic pulvinar and LGN (Fig. 6A, Table 2) (Wilcoxon rank-sum tests, Bonferroni corrected, V4-vPul: p = 4.19e-11; V4-dPul: p = 7.08e-11, V4-LGN: p = 1.00e-06). Albeit firing rates tended to be lower in the pulvinar as compared to both V4 and LGN (Fig. 6B), the mean firing rates during the examined interval were not statistically different after Bonferroni correction (Wilcoxon rank-sum tests, V4-vPul p = 0.33; V4-dPul p = 0.03; V4-LGN p = 0.99; vPul-LGN p = 0.33; dPul-LGN p = 0.01; vPul-dPul p = 0.16). Generally, we found trial-to-trial variability to increase with the size of the counting window. Figure 6C shows the mean Fano factor across populations as a function of window size (10–200 ms), indicating that neural spiking in cortical area V4 was more variable across trials than in the thalamic populations independently of the size of the window in which spikes were counted.

**Table 2:** Mean Fano factors and firing rates +/− standard deviation during ongoing activity 1000–500 ms prior to target stimulus onset. N denotes the number of recording sites.

N	V4	vPul	dPul	LGN
	100	118	136	105
mean Fano factor +/− SD	1.80 +/− 0.93	1.17 +/− 0.29	1.25 +/− 0.58	1.30 +/− 0.48
mean firing rate (spikes/s) +/− SD	46 +/− 65	38 +/− 62	32 +/− 36	46 +/− 61

Moreover, trial-to-trial variability during initial fixation and the magnitude of subsequent variability quenching following stimulus onset was significantly correlated on a site-by-site basis in V4 (Pearson’s correlation coefficient r = −0.49, p = 1.74e-7), but not in the thalamic regions that did not show significant quenching (Pearson’s correlation coefficients, vPul: r = −0.14, p = 0.11; dPul: r = −0.11, p = 0.17; LGN: r = −0.06, p = 0.51). These results suggest that even prior to the onset of a visual stimulus sizeable differences exist between cortical and thalamic spiking variability that may impact subsequent quenching behaviour.

## Discussion

As expected from previous studies^1,30,31,36^ visual stimulation significantly reduced trial-to-trial variability in area V4. In the pulvinar and LGN, we found the variability quenching effect to be either absent or, in case of the dorsal pulvinar, marginal compared to the well-described variability decline in cortex, even when firing rate distributions were matched. In the ventral portion of the pulvinar, which exchanges recurrent connections with extrastriate cortex including area V4, the quenching effect was entirely absent despite it likely receiving a large portion of inputs from V4.

While we observed a slightly lower response variability in the pulvinar compared to visual cortex, spiking activity in LGN and pulvinar was also significantly less variable than in area V4 during steady fixation when visual input did not change. This comparatively reliable activity in the thalamus could in theory account for the lack of stabilization by stimulus onset as spiking variability is already low even when the cells are not driven by external input and may simply not be further reduced due to natural constraints underlying spike generation. One of the simplest models of neural spiking is a Poisson process, which is characterized by spikes occurring independently of each other, resulting in the spike count variance being equal to the mean (Fano factor = 1). Although low or even sub-Poisson Fano factor values have been measured in some instances^28^, cortical spiking is typically reported to show a considerable amount of additional variance, which can largely be explained by excitability fluctuations that are correlated over time and between neurons^10,37^. Assuming that spiking noise is approximately Poisson, there may be an irreducible lower bound to variability in neural systems that limits the impact of external input on neural populations that inherently show little excess variance^8^. Since neural activity likely arises from an interaction between ongoing activity and stimulus-driven responses, it is possible that the pulvinar does not exhibit substantial quenching behaviour because variability is negligible even before stimulus onset. The fact that the V4 sites that were least variable prior to stimulus onset also showed the smallest magnitude of quenching supports this view.

Previous studies proposed that the stimulus-induced reduction of firing rate variability may constitute a general property of large recurrent networks^1^ and can naturally arise from a pattern of balanced inhibition and excitation in an attractor network^38^. While the network switches between multiple attractor states as long as it is not externally driven, excitatory input stabilizes one specific attractor and thus reduces spiking variability within the network. Given that both LGN and pulvinar do not show an equivalent decrease in variability, it is possible that differences in local network architecture between thalamus and cortex explain the stable activity within the thalamus. For example, while neocortical neurons are extensively interconnected in a highly specific manner^39^, there is no evidence for direct connections between relay cells in the thalamus^16^.

We observed a small but significant quenching effect in the dorsal pulvinar in response to the target stimulus which was followed by a hand movement, but not to the RDM stimulus which did not prompt a behavioural response by the animal. Closer inspection revealed no significant difference in trial-to-trial variability before and after the movement. However, variability changes in premotor cortex have been shown to occur following relevant visual cues but prior to the movement^34^, suggesting that the quenching effect we observed in the dorsal pulvinar may have nonetheless been related to motor preparation as well.

Our results are consistent with an earlier study that found response variability in the pulvinar to be lower than in extrastriate visual cortex V4, although no comparisons of stimulus-induced variability changes were made^27^. Interestingly, the authors reported modulations of excitability associated with attentive fixation that occurred in both pulvinar and cortex and were of similar magnitude when variability differences between regions were taken into account. We did not manipulate attentional state in the current study, but it can be assumed that similar modulations of excitability occurred during the fixation period prior to the onset of the target stimulus that prompted a response by the animal. In addition to modulations of firing rate, attention has been associated with reduced trial-to-trial variability of V4 responses compared to unattended stimuli as well as during sustained attention^30,36^ and the magnitude of attentional modulation in the pulvinar has been found to be significantly smaller than in area V4^26^ so it is likely that these differences in modulation strength and the differences in the degree of variability between cortex and pulvinar during fixation are closely linked.

In addition, experimental evidence from rodents and primates suggests that the functional role of higher-order thalamic nuclei such as the pulvinar and the mediodorsal thalamus goes beyond that of a mere cortico-cortical relay and is critical for the flexible coordination of activity within and across cortical regions^15,23,26,40,41^. In particular, the mediodorsal pulvinar has recently been shown to coordinate the fronto-parietal network directing spatial attention in macaques^40^. These findings are consistent with the view that the pulvinar and mediodorsal thalamus contain circuits that, rather than transmitting information from one cortical area to another, shift and sustain functional connectivity across cortex according to task demands^15^.

Response variability in visual cortex has been shown to strongly depend on cortical state as excitability fluctuations that account for much of the variability in cortical responses as well as spontaneous activity are largest in synchronized states^10^. The significantly lower variability in the pulvinar compared to V4 we observed suggests that the impact of these fluctuations on spiking activity in the pulvinar are minimal despite its extensive reciprocal connections with the cerebral cortex.

## Materials and Methods

Part of the data from pulvinar and LGN contain neurons and multi-unit data that have been included in a previous paper that focused on average firing rates during perceptual suppression^24^. Part of the V4 data set has been included in a paper on the features of visual adaptation in area V4^42^. The previous studies did not investigate neural variability. All experiments were approved by and conducted following the guidelines of the National Institutes of Health (Bethesda MD, USA).

### Electrophysiological recordings

We recorded multi-unit (MUA) and single-unit (SUA) activity from ventral area V4 (81 MUA, 19 SUA), the thalamic pulvinar (dorsal pulvinar: 102 MUA, 34 SUA; ventral pulvinar: 108 MUA, 12 SUA) and the LGN (89 MUA, 16 SUA) in two adult Rhesus macaques (*Macaca mulatta*, monkey E and B). Data were recorded during a total of 113 recording sessions (62 monkey E, 51 monkey B) with 4 to 8 microelectrodes simultaneously (Thomas Recording GmbH, Giessen, Germany). Of those sessions, 95 recordings (49 monkey E, 46 monkey B) contained sites that met our inclusion criteria (uninterrupted recordings without drifts, a minimum number of 10 identical trials as well as a minimum mean firing rate of 5 spikes/s) and were considered for analysis. Spiking activity was collected with the MAP recording system (Plexon Inc., Dallas TX, USA). Spike candidates were identified online through RASPUTIN software (Plexon Inc., Dallas TX, USA) on a PC receiving the digitized signals. Waveform thresholds were manually adjusted for each channel before the start of data acquisition. After the experiment, units were isolated in PCA space in the commercially available “Offline Sorter” (Plexon Inc., Dallas TX, USA) and time stamps were saved for further analysis.

### Reconstruction of recording sites

Surgical procedures, methods and details of reconstruction of the thalamic sites are described in^24^ and for V4 in^42^. In short, recording sites were identified on the basis of chamber coordinates, using MRI scans with gadolinium filled chamber grids. For the thalamic recordings, LGN was used as an additional reference point, which was identified on the basis of its monocular responses in combination with small receptive field sizes. There are multiple parcellation schemes available for the pulvinar^43,44^. In the absence of histology but equipped with high resolution 4.7T MRI, we coarsely separated the pulvinar into a ventral and a dorsal portion, using the brachium of superior colliculus as division line, which is well visible on the MRI scans and has been used in previous studies^19,22,45,46^. The dorsal pulvinar (dPul) includes the medial pulvinar and the dorsal part of the lateral pulvinar (also denoted as PLdm, or Pdm in earlier papers (e.g.^47^), whereas the ventral pulvinar contains the inferior pulvinar and ventral part of lateral pulvinar (also denoted as PLvl)^48,49^. The majority of the reported dorsal pulvinar sites were recorded from the dorsal part of the lateral pulvinar^24^.

### Stimuli and task

Electrophysiological data were collected in the context of a Generalized Flash Suppression (GFS) paradigm^24^. Stimuli were displayed on 38 × 65 cm monitors through a mirror stereoscope. The screen to eye distance was 88 cm. A small fixation spot (0.15°) was always presented in the middle of the screen and monkeys were required to maintain fixation within a radius of 0.7° visual angle. Monkeys were required to fixate for 1500 ms before the target stimulus appeared at a parafoveal position. Target stimuli consisted either of a red luminance patch or grating of 0.3° to 6° visual angle and the eccentricity of the targets (0.3°–7.7°) was varied on a session basis depending on the position of the receptive fields (RF) of the best isolated recorded neurons. Following target onset, monkeys had to pull a lever.

### Data analysis

Neurophysiological data were processed and analysed offline using custom-written software in MATLAB 2015b (The MathWorks Inc., Natick MA, USA). Data from the two animals were similar (Supplementary Information Fig. S1) and are thus considered together. The Fano factor was calculated as the variance divided by the mean of spike counts for each recording site and interval of interest. For graphical representation of trial-to-trial variability over time the Fano factor was computed using a 50 ms sliding window moving in 10 ms steps and then smoothed using a moving average. For statistical analysis, the Fano factor was calculated in non-overlapping 50 ms windows covering the respective trial interval and averaged to obtain a single value per interval.

For the analysis of stimulus-driven variability changes we considered target stimuli that were presented to the left eye, which was the ocular configuration that yielded the greatest number of identical trials. The mean number of trials in a given session for this condition was 93 for V4, 82 for ventral pulvinar, 82 for dorsal pulvinar and 85 for LGN sites. We found both V4 and thalamic responses to be well captured by a 300 ms window following stimulus onset. We compared Fano factors of the 300 ms pre and 300 ms post target stimulus intervals and calculated percent change scores for the Fano factor decline with stimulus onset according to (FF post - FF pre)/FF pre for each recording site (V4 N = 100, vPul N = 118, dPul N = 136, LGN N = 105) to quantify the magnitude of quenching.

Statistical significance was assessed with Wilcoxon signed-rank tests or Wilcoxon rank-sum tests for comparisons between brain regions due to the fact that raw Fano factor values were not normally distributed (Shapiro-Wilk test, p > 0.05). We used one-sample as well as two-sample t-tests for the normally distributed percent change values. We categorically applied a Bonferroni correction for multiple comparisons and used a significance level of 0.05/4 = 0.0125 for one-sample tests within regions and a significance level of 0.05/6 = 0.0083 for two-sample tests comparing effects between regions.

Significant responses to the visual target stimuli were determined by Wilcoxon rank-sum tests (p < 0.05) using a 100 ms windows immediately preceding stimulus onset and at a latency between 50 and 150 ms after stimulus onset. To determine the contribution of rising and falling rates on the variability decline following stimulus onset, we calculated the Pearson’s correlation coefficient between changes in Fano factor and the absolute visually evoked response for sites that showed significant responses.

To correct for differences in firing rates we used the distribution-matching procedure developed in a previous study^1^. Specifically, we calculated mean spike counts of each site in all 12 non-overlapping 50 ms windows spanning the 300 ms pre to 300 ms post stimulus period for each region separately. We then determined the greatest common firing rate distribution at a bin resolution of 5 spikes/s across all pre- and post-stimulus time points for each region. We randomly selected subpopulations of sites yielding the same bin heights as the determined greatest common firing rate distribution so as to simulate a constant mean firing rate over time. Based on these sites we then calculated the Fano factor for each time window and averaged the results across 1000 repetitions of random selection of suitable data points.

We further analysed a trial period of stable fixation without changes in the visual input 1000–500 ms prior to target presentation for which we pooled the available trials which were identical across the whole session, thus obtaining a mean number of 178 trials for V4, 161 for the ventral pulvinar, 164 for the dorsal pulvinar and 170 for the LGN. We calculated the average Fano factor for each recording site (V4 N = 100, vPul N = 118, dPul N = 136, LGN N = 105) as a function of the window size in which spikes were counted (10–200 ms in steps of 10 ms) as well as in non-overlapping 50 ms windows for statistical comparison. Finally, we calculated the Pearson’s correlation coefficient between the average Fano factor during 1000–500 ms prior to target interval and the subsequent percent change in Fano factor with stimulus onset.

## Supplementary information

Supplementary Information

## Data Availability

The datasets analyzed during the current study are available from the corresponding author on reasonable request.
