# Author Correction: Thalamus exhibits less sensory variability quenching than cortex

**DOI:** 10.1038/s41598-020-59858-8

**Published:** 2020-02-19

**Authors:** Eva Poland, Tobias H. Donner, Kai-Markus Müller, David. A. Leopold, Melanie Wilke

**Affiliations:** 1 Department of Cognitive Neurology, UMG, University Medicine Goettingen, Robert-Koch-Str. 40, Goettingen, 37075 Germany; 2 University Medical Center Hamburg-Eppendorf, UKE, Department of Neurophysiology and Pathophysiology, Building N43, Martinistr. 52, 20246 Hamburg, Germany; 3 Consumer Behavior, HFU Business School, Jakob-Kienzle-Str. 17, 78054 Villingen-Schwenningen, Germany; 4 Section on Cognitive Neurophysiology and Imaging, Laboratory of Neuropsychology, National Institute of Mental Health, National Institutes of Health, Building 49, Room B2J-45, MSC-4400 49 Convent Dr., Bethesda, MD 20892 USA; 5 DfG Center for nanoscale Microscopy & Molecular Physiology of the Brain (CNMPB), Robert-Koch-Str. 40, Göttingen, 37075 Germany; 6 German Primate Center, DPZ, Leibniz Institute for Primate Research, Kellnerweg 4, Goettingen, 37077 Germany

Correction to: *Scientific Reports* 10.1038/s41598-019-43934-9, published online 20 May 2019

The original version of this Article contained errors in the spelling of the authors Eva Poland, Tobias H. Donner, Kai-Markus Müller, David. A. Leopold & Melanie Wilke which were incorrectly given as E. Poland, T. H. Donner, K. –M. Müller, D. A. Leopold & M. Wilke respectively.

These errors have now been corrected in the PDF and HTML versions of the Article, and in the accompanying Supplementary Information file.

