# Supplementary material for: Thalamus exhibits less sensory variability quenching than cortex
Source: Sci Rep. 2019 May 20;9:7590. doi: 10.1038/s41598-019-43934-9 (PMC6527544; doi:10.1038/s41598-019-43934-9)
Supplement: Supplementary file 1 — Supplementary Information [file 41598_2019_43934_MOESM1_ESM.pdf]

Thalamus exhibits less sensory variability quenching than cortex

E. Poland[1], T. H. Donner[2], K.-M. Müller[3], D. A. Leopold[4], M. Wilke[1,5,6]

SUPPLEMENTARY INFORMATION

S1 Separate results of the two animals

The main results of the current study, namely (1) the lack of quenching in the thalamic regions compared to cortical area V4, as well as (2) the significantly lower levels of trial-to-trial variability in the thalamic regions absence of changes in stimulus input, were remarkably similar between the two animals. The mean percent change in Fano factor with the onset of the target stimulus for the two monkeys is illustrated in **Figure S1A**. Both animals showed an individually significant quenching effect in area V4 (One-sample t-tests on the percent change in Fano factor with stimulus onset, monkey E: p = 2.23e-11, N = 85, monkey B: p = 0.01, N = 15). A significant decline in Fano factor in the dorsal pulvinar was also observed in both cases (One-sample t-tests, monkey E: p = 1.70e-04, monkey B: p = 0.03), while the decreases were significantly smaller than those in V4 (Two-sample t-tests, monkey E: p = 2.42e-3, monkey B: p = 0.01). A quenching effect was consistently absent in the ventral pulvinar portion (One-sample t-tests, monkey E: p = 2.23e-11, N = 72, monkey B: p = 0.01, N = 48) as well as in the LGN (One-sample t-tests, monkey E: p = 2.23e-11, N = 45, monkey B: p = 0.01, N = 60). **Figure S1B** shows the average Fano factors during the fixation period 1000 – 500 ms prior to target onset for both animals separately. The statistical comparisons between area V4 and all thalamic nuclei (vPul, dPul, LGN) were consistent between and individually significant in both monkeys (Wilcoxon rank-sum tests, V4-vPul: monkey E p = 2.13e-06, monkey B p = 2.07e-05; V4-dPul: monkey E p = 4.18e-04, monkey B p = 1.81e-05; V4-LGN: monkey E p = 1.40e-05, monkey B p = 0. 35e-2), confirming the significantly lower variability in the thalamic regions compared to cortical area V4 prior to stimulus onset.

S2 Comparison of single- and multi-unit data

Single- and multi-unit activity were pooled for the current study as they were generally very similar (**Figure S2A-B**). Restricting the analysis of variability changes with target onset to the few available single units confirmed the significant decrease in area V4, while it was absent in dorsal and ventral pulvinar as well as LGN cells (One-sample t-tests on the percent change in Fano factor with stimulus onset, V4 N = 19, p = 4.00e-3, vPul N = 12, p = 0.33, dPul N = 34, p = 0.06, LGN N = 16, p = 0.39). Similar results were obtained for the multi-unit data (One-sample t-tests, V4 N = 81, p = 8.64e-11, vPul N = 108, p = 0.90, dPul N = 102, p = 8.69e-04, LGN N = 89, p = 0.12). Trial-to-trial variability during stable fixation was slightly lower in the single-unit data of V4 and the dorsal pulvinar than in the respective multi-unit activity, but there was no significant difference (Wilcoxon ranked-sum tests, V4: p = 0.41, dPul: p =0.69).

S3 Shared variability analysis

We wondered to which degree the trial-to-trial variability we observed was shared between neurons and examined simultaneously recorded unit pairs within area V4 and the pulvinar (V4-V4 N = 141, vPul-vPul N = 201, dPul-dPul N = 174) as well as between V4 and the pulvinar (V4-vPul N = 117, V4-dPul N = 109, vPul-dPul N = 28). For these pairs, we calculated the spike count correlations rSC (sometimes also referred to as noise correlations) as the Pearson's correlation coefficient of spike counts for the 300 ms pre and the 300 ms post target stimulus intervals. Within area V4, shared variability decreased significantly from pre to post stimulus interval (Wilcoxon signed-rank test, p = 5.29e-10, **Figure S3A**). Within the dorsal and ventral pulvinar we did not observe any significant changes in spike count correlations with stimulus onset (Wilcoxon signed-rank tests, vPul-vPul p = 0.73; dPul-dPul p = 0.91; **Figure S3A)**. There was no significant decrease in shared variability between regions (Wilcoxon signed-rank tests, V4-vPul p = 0.52; V4-dPul p = 0.94; vPul-dPul p = 0.62; **Figure S3B**).

S4 Changes in microsaccade rate with stimulus onset

Changes in neural trial-to-trial variability have previously been associated with small eye movements (microsaccades) that occur during fixation [1]. We thus aimed to assess the influence of microsaccades on the quenching effect. Consistent with a previous study[2], we found the microsaccade rate to decrease following the onset of the target stimulus (**Figure S4A,** 300 ms pre to 300 ms post target onset, Wilcoxon signed-rank test, $p = 2.96e-10$). To investigate whether the stimulus-induced variability reduction in V4 and dorsal pulvinar could be attributed to changes in microsaccade rate we calculated the Fano factor for trials that did not contain microsaccades in the relevant trial period -300 ms to 300 ms relative to target stimulus onset. In those microsaccade-removed trials a significant decrease of variability following target onset was still obtained in area V4 but not in either pulvinar subnucleus nor in the LGN (**Figure S4B**, Wilcoxon signed-rank tests, V4 $p = 6.05e-09$, dPul $p = 0.39$; vPul $p = 0.58$; LGN $p = 0.30$).

References

1. Gur, M., Beylin, A. & Snodderly, D. M. Response variability of neurons in primary visual cortex (V1) of alert monkeys. *J. Neurosci. Off. J. Soc. Neurosci.* **17,** 2914–2920 (1997).
2. Cui, J., Wilke, M., Logothetis, N. K., Leopold, D. A. & Liang, H. Visibility states modulate microsaccade rate and direction. *Vision Res.* **49,** 228–236 (2009).

Figure S1

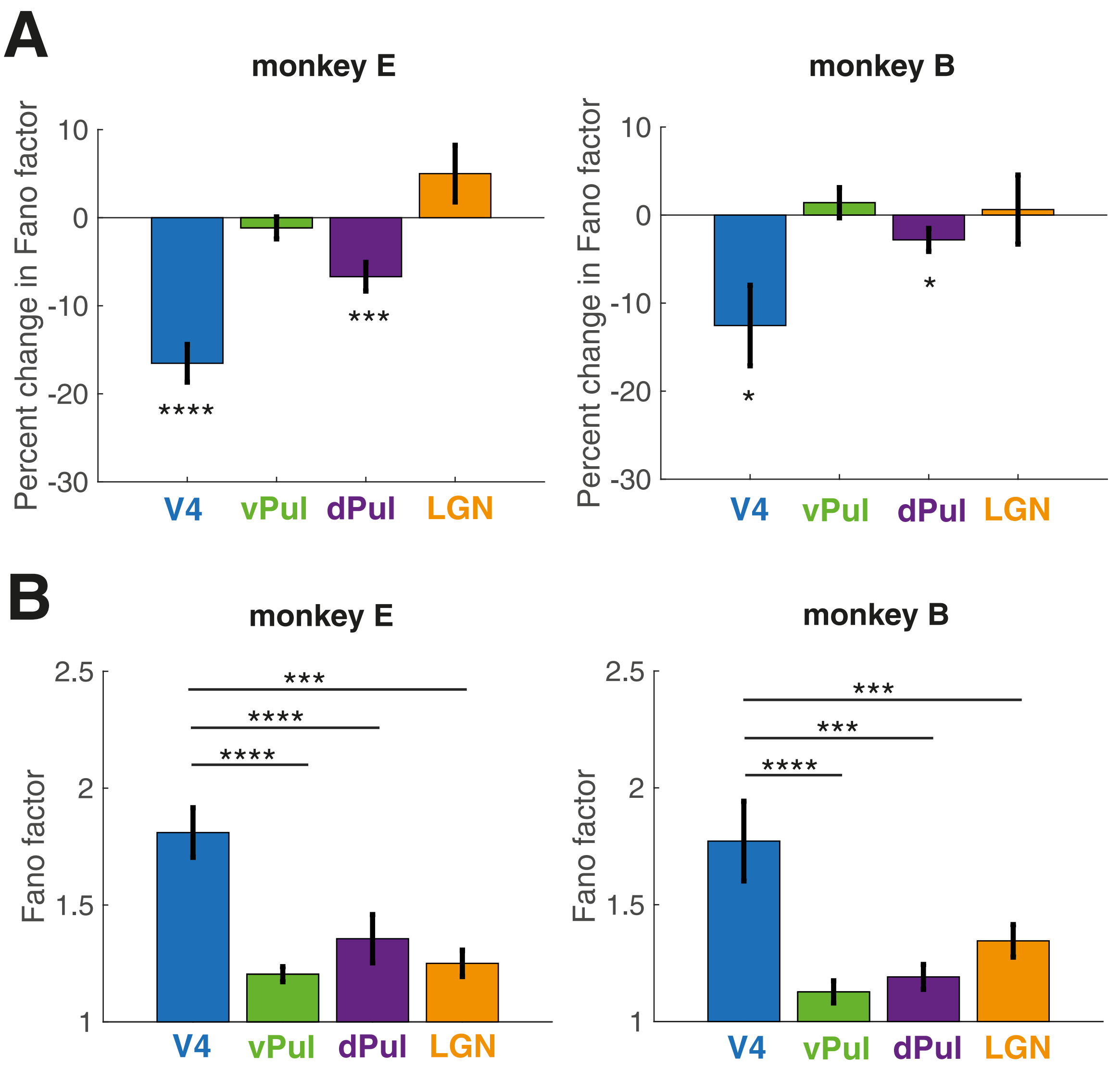
A
monkey E
Percent change in Fano factor
10
0
-10
-20
-30
****
***
V4
vPul
dPul
LGN
monkey B
Percent change in Fano factor
10
0
-10
-20
-30
*
*
V4
vPul
dPul
LGN
B
monkey E
Fano factor
2.5
2
1.5
1
***
****
****
V4
vPul
dPul
LGN
monkey B
Fano factor
2.5
2
1.5
1
***
***
****
V4
vPul
dPul
LGN

Figure S2

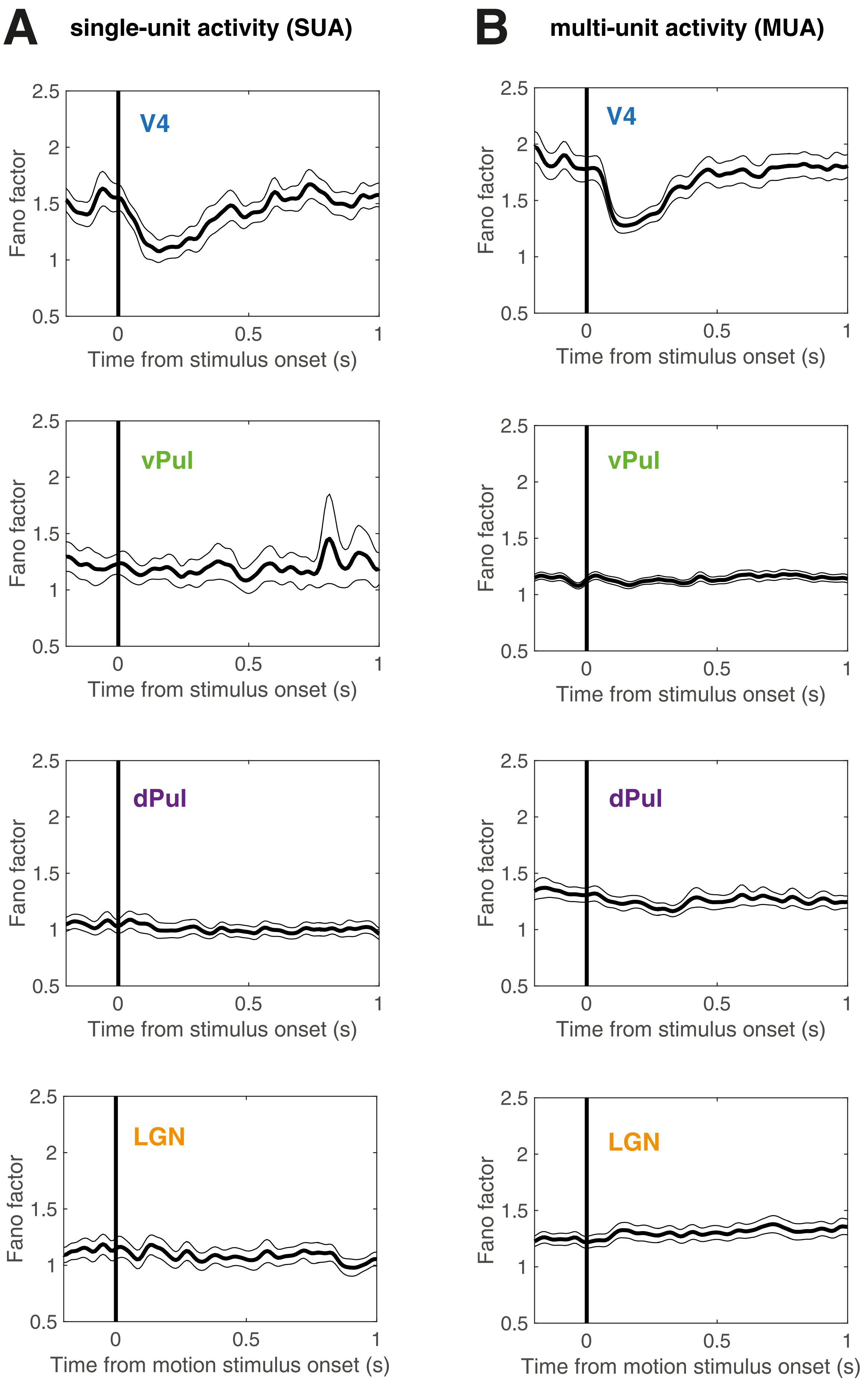
A
single-unit activity (SUA)
B
multi-unit activity (MUA)
V4
vPul
dPul
LGN
Fano factor
2.5
2
1.5
1
0.5
0
0.5
1
Time from stimulus onset (s)
Time from motion stimulus onset (s)

Figure S3

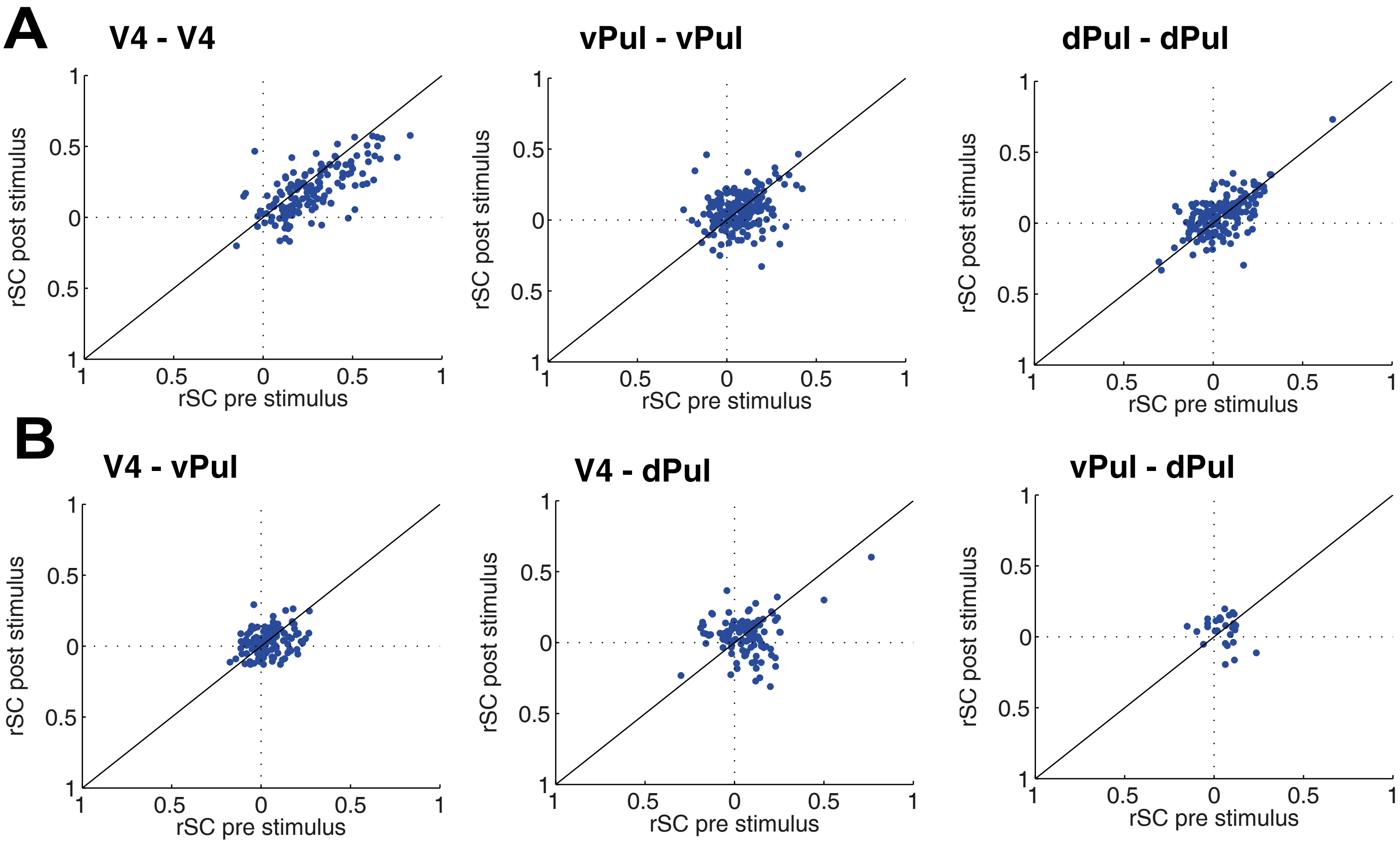
A
V4 - V4
vPul - vPul
dPul - dPul
B
V4 - vPul
V4 - dPul
vPul - dPul
rSC post stimulus
rSC pre stimulus
1
0.5
0
0.5
1

Figure S4

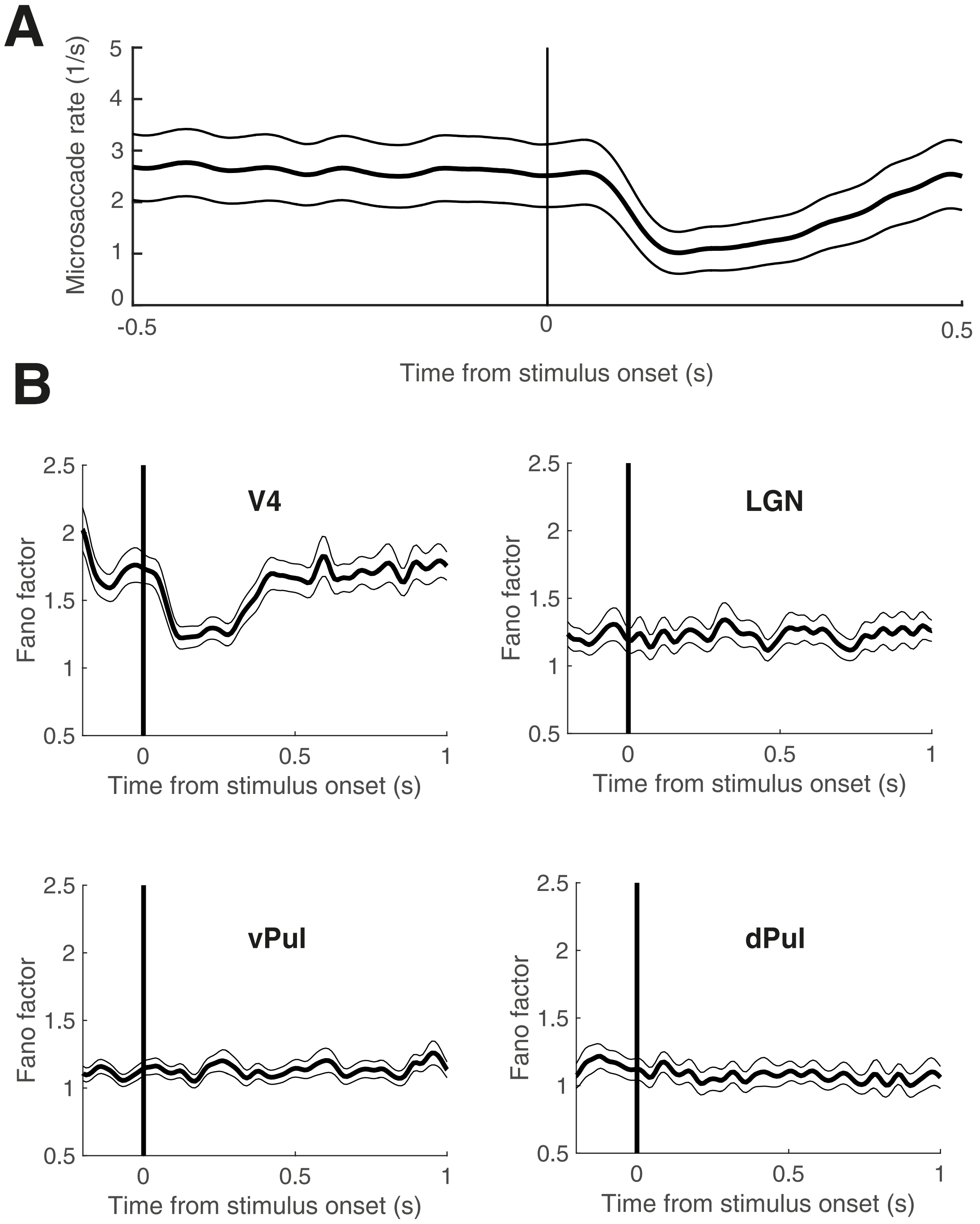
A
Microsaccade rate (1/s)
5
4
3
2
1
0
-0.5
0
0.5
Time from stimulus onset (s)
B
V4
Fano factor
2.5
2
1.5
1
0.5
0
0.5
1
Time from stimulus onset (s)
LGN
Fano factor
2.5
2
1.5
1
0.5
0
0.5
1
Time from stimulus onset (s)
vPul
Fano factor
2.5
2
1.5
1
0.5
0
0.5
1
Time from stimulus onset (s)
dPul
Fano factor
2.5
2
1.5
1
0.5
0
0.5
1
Time from stimulus onset (s)
